# Chemical Composition and Sensory Evaluation of Saffron

**DOI:** 10.3390/foods10112604

**Published:** 2021-10-27

**Authors:** Stefano Predieri, Massimiliano Magli, Edoardo Gatti, Francesca Camilli, Pamela Vignolini, Annalisa Romani

**Affiliations:** 1 IBE-CNR, Institute of BioEconomy, c/o Area della Ricerca di Bologna, Via P. Gobetti, 101, 40129 Bologna, BO, Italy; stefano.predieri@ibe.cnr.it (S.P.); massimiliano.magli@ibe.cnr.it (M.M.); edoardo.gatti@ibe.cnr.it (E.G.); 2 IBE-CNR, Institute of BioEconomy, Via Caproni 8, 50145 Firenze, FI, Italy; francesca.camilli@ibe.cnr.it; 3 Phytolab, Department of Statistics, Informatics, Applications “G.Parenti”, DiSIA, University of Florence, Via Ugo Schiff 6, 50019 Sesto Fiorentino, FI, Italy; annalisa.romani@unifi.it

**Keywords:** saffron, picrocrocin, safranal, descriptive sensory analysis, HPLC/DAD (High Performance Liquid Chromatography) analysis

## Abstract

The quality and economic value of saffron, one of the most counterfeited spices, are based on three key substances that are relatively easy to measure: crocines (colour); picrocrocines (bitter); safranal (odour impact). Despite being well-known, as their concentration is correlated to sensory intensity, a detailed sensory evaluation, performed by a trained panel, supported by advanced analytical approaches, may better show the relationships between saffron composition and sensory perception. Three saffron samples of different Italian origins (Sardinia and Tuscany) were evaluated by a trained sensory panel and their chemical composition was determined by HPLC (High Performance Liquid Chromatography) and spectrophotometry. Safranal concentration and the perceived odour intensity were positively correlated while relationships between picrocrocine and bitter perception were more complex to detect. By correlating (Multiple Factor Analysis) saffron sensorial and chemical profiles, this work aims at improving saffron characterisation while providing better information on the quality of this valuable spice.

## 1. Introduction

Saffron is the spice obtained from the stigmas of *Crocus sativus* L., a perennial stemless herb of the Iridaceae family, widely cultivated in Iran and other countries, including India and Greece [1], but also Spain [2] and Italy [3] where its cultivation has longstanding traditions and yields high-quality productions.

It is well known that saffron is one of the most valuable spices in the world, traditionally used in many food preparations, but also appreciated for its properties in traditional medicine as many ethnobotanical studies report [4,5,6,7]. In addition, several types of research refer to interesting pharmacological applications of saffron and its effects on several pathologies [8,9,10,11,12,13,14,15,16]. This spice is commonly grown in relatively small and definite geographic areas which provide it with peculiar biochemical traits [17,18,19,20]. Saffron composition is known to affect sensory perception and consumer appreciation. Compounds such as crocins (glycoside derivatives of crocetin) are associated with colour intensity; picrocrocin is addressed as the major responsible for the bitter taste [21] while safranal (monoterpene aldehyde) provides the typical saffron flavour. Sensory evaluations, based on judgments of trained panellists, along with consumer studies, may provide useful information about correlations between saffron chemical composition and sensory evaluation. Reports of a whole product evaluation performed by a trained panel are scarce. Sánchez et al., 2011 [22], focused on the bitterness perception of saffron and its extracts, while Chrysanthou et al. (2016) [21] conducted wide research on pure picrocrocin detection thresholds and the factors affecting them (temperature, other constituents, ethanol content). When testing picrocrocin in presence of crocetin esters, a masking effect of this major class of saffron constituents affecting bitter taste perception was reported [21]. Furthermore, as saffron is the most counterfeited spice, the EU (European Union) has recently introduced an ISO standard (ISO/TS 3632-2:2003) considering saffron as a food. According to such standard, the inclusion, in the commercial label, of secondary metabolites, in addition to minerals and nutrients, is mandatory.

Tuscany and Sardinia, the regions considered in this study, are two of the most saffron productive areas in Italy (the first one is Abruzzo). The importance of the saffron economy in these regions is attested by the two PDO (Protected Designation of Origin)labels of saffron produced in San Gimignano, in Tuscany, and San Gavino, Turri and Villanovafranca in Sardinia [23]. As reported by Sonnino, 2007 [24] in Tuscany saffron is a spice deeply rooted in gastronomic traditions since the Middle Ages. Thus, its value is both economic and cultural and can be a significant development potential for producers. In Italy food products appear to be forever rooted in a particular place. The PDO labels are very important from both an economic and cultural point of view [25].

Furthermore, the protection granted by the EU represents a tool to protect this product against misuses and frauds, but also its reputation being locally focused can better activate and reinforce rural development dynamics [26]. In Sardinia, the PDO label acknowledgement has pushed also other areas to cultivate this spice.

Thus, the objectives of this study were (i) to provide a more comprehensive characterisation of Italian saffron through a detailed sensory evaluation, performed by a trained panel and correlated with advanced analytical results; (ii) to improve the Italian saffron characterisation by evaluating nutritional parameters.

To achieve that, a preliminary study on three Italian samples of saffron of different geographical origins (Sardinia and Tuscany) were analysed crocins, safranal, picrocrocins and flavonols were identified by HPLC-DAD analyses of the hydroalcoholic (EtOH:H_2_O 70:30, pH 3.2) extracts. A sensory analysis was also conducted by a trained panel on the three saffron samples and particular attention was dedicated to components interactions. A specific analysis of the sensory properties of crocins was performed.

## 2. Materials and Methods

### 2.1. Saffron Samples and Preparation

Three saffron samples from three areas of two Italian regions (Sardinia: sample SA; and Tuscany: sample CT from central Tuscany and sample GR from South Tuscany) were analysed.

Saffron water extracts were prepared infusing 5 mg of dried stigmas, from each sample, in 10 mL of water. The solutions were filtered in order to obtain the extracts to be analysed by HPLC/DAD for the determination of saffron components.

Preliminary sensory tests defined the tasting protocol: 100 mg/100 cc of saffron, hydrolysed in low mineralised water at 80 °C for 60 min, cooled at 24 ± 2 °C. Tests were performed within two hours of preparation. Authentic standards of crocin (Fluka, St. Louis, MO, USA), ranging from 0.94 to 60 ppm, were used as reference solution.

### 2.2. HPLC/DAD Analysis

Polyphenols were identified using data from HPLC/DAD analyses by comparing and combining their retention times and UV/Vis spectra with those of authentic standards and data reported in a previous work [27]. Quantification of individual compounds was directly performed by HPLC/DAD using a five-point regression curve (r^2^ ≥ 0.998) in the range 0–30 μg on the basis of authentic standards. In particular, crocin derivatives were determined at 440 nm using curcumin as reference compound. Flavonols were determined at 350 nm using quercitrin as reference compound, picrocrocin was determined at 280 nm using cinnamic acid as reference compound and safranal was determined at 308 nm using safranal as reference compound. In all cases, real concentrations of the derivatives were calculated after applying, if possible, corrections for differences in molecular weight.

### 2.3. Nutritional Table

Parameters, such as, moisture, ash, fibres, proteins, fat and sugars were also evaluated with the main analytical methods used by the Istituto Superiore di Sanità (Italian National Institute of Health www.iss.it (accessed on 15 November 2020).) for food chemical control. Microelements concentration were determined using method Plant Tissue 1 from CEM microwave (Mars Xpress; max power 1200 W, Power 100%, Ramp time 15 min, T 200 °C, hold time 15 min) for digestion. All samples were then analysed by means of iCAP™ 7400 ICP-OES Analyzer (Thermofisher Iris Intrepid II—SelectScience^®^ Bath, UK). ICP-AES measurements were preceded by a calibration step using aqueous mixed standards.

### 2.4. Descriptive Sensory Analysis

A panel of judges (12), proficient in sensory evaluation, was trained at evaluating saffron by descriptive analysis (DA) conducted using a structured 9 point scale (1: non perceptible; 5: medium intensity; 9: extremely intense). A list of descriptors list was developed, based on literature [28] and implemented with attributes, further defined during the sensory focus groups, conducted using flash profile techniques [29]. The evaluated attributes were: Odour intensity, Safranal odour, Fruity odour, Flowery odour, Grassy odour, Bitterness, Astringency, Pungency, Flavour intensity, Safranal flavour, Fruity flavour, Grassy flavour, Starch flavour, Other odour and flavours. The correctness of the attribute list was validated by proposing saffron of different quality, origin and concentration. The training was integrated by the evaluation of substances representative of basic saffron components such as safranal (either natural or synthesised), picrocrocin and crocins. Crocins perception threshold was also studied according to the method reported in Crisanthou et al. [21]. Saffron evaluations were performed at the sensory lab facility of the Institute for BioEconomy of the National Research Council (Bologna, Italy) in individual booths under white light. Trained panel participated in two sessions; the three samples were evaluated in each session (for a total of two replicates). Samples were evaluated monadically. The order of the sample presentation was randomised among assessors using a balanced Latin square design. Saffron samples (30 mL) were presented in plastic cups identified by a three-digit code.

### 2.5. Statistical Analysis

In this study two sets of variables, sensory and instrumental data, were considered and multiple factor analysis (MFA) was used to correlate them. MFA, applied in several sensory studies, is a very well-established method dealing with sets of individuals described by several sets of variables [30].

Sensory attribute intensity data collected were submitted to a 3-way ANOVA (Analysis of Variance) model (main factors: samples, replicates and assessors) with interactions, significance level fixed at *p* ≤ 0.05. Concerning the sensory attribute intensity differences between samples, it was assessed by applying the Tukey test for multiple comparison of means, taking into account the following 0.05, 0.01, 0.001 F-values for significance levels.

## 3. Results

The profile of Italian saffron of three different geographical origins was characterised both from a biochemical and sensory analytical point of view, as reported in the following paragraphs.

### 3.1. Chemical Profile of Saffron Samples

Data obtained from the HPLC/DAD analysis of the dried stigmas are reported in Table 1.

The main set of detected compounds were crocins analogues, the major biologically active components of saffron [31]. They are all glycosides of trans-crocetin, a carotenoid derivative which is responsible for saffron colour. The results show that the three samples differed in trans-crocin 4 (169.97 ± 2.18, SA; 405.19 ± 3.95, CT; 249.82 ± 2.92, GR), trans-crocin 3 (61.22 ± 1.80, SA; 132.07 ± 1.98, CT; 93.90 ± 2.15, GR) and picrocrocin (74.33 ± 1.92, SA; 112.62 ± 2.01, CT; 29.13 ± 1.01, GR) contents. Such compounds were also detected in larger amount in each saffron sample. In particular, the CT sample showed the highest content in crocins and picrocrocin, giving evidence of the very good quality of the product.

Safranal (2,6,6-trimethyl-1,3-cyclohexadien-1-carboxaldehyde), responsible for the characteristic aroma of saffron, is formed during the storage by dehydration of picocrocin which provides the spice with the characteristic bitter taste. The highest content of safranal was found in SA and GR saffron.

Parameters, such as, moisture, ash, fibres, proteins, fat, sugars and minerals (Na, K, Mg, Ca, Fe, C, Zn, Mn) were also evaluated to draft a nutritional table aimed at developing for the label of commercial saffron (Table 2). The nutritional table shows that microelements are the most representative class of saffron. Among them, K is the most represented element in all the samples analysed.

### 3.2. Sensory Profile of the Saffron Samples

**Figure 1:**
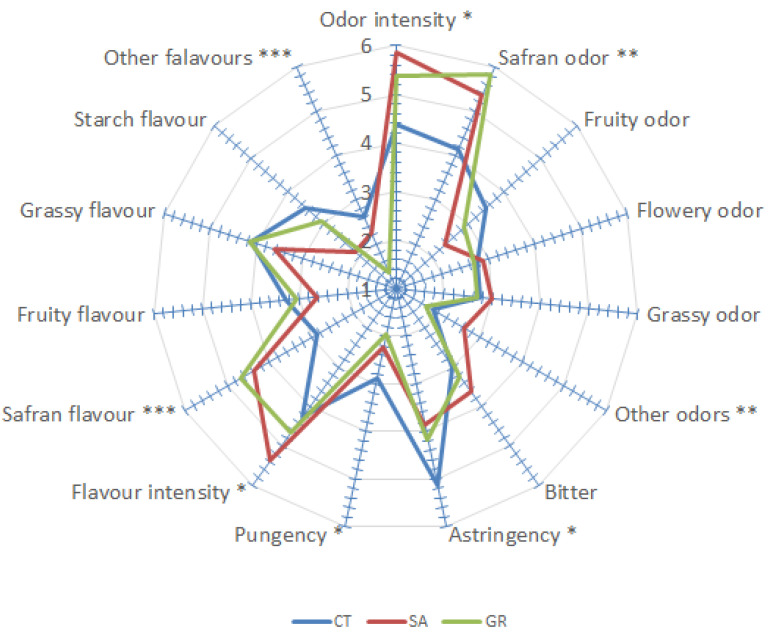
Attributes intensity as defined by panel thresholds. Attributes found to be significantly different (Tukey’s HSD test) are indicated by *, **, ***, referred to significant F-values: * 0.05, ** 0.01 or *** 0.001 levels.

The results of sensory analysis applied to SA, CT and GR saffron samples are reported in Figure 1 and Table 3.

Significant differences were found for overall intensity (Figure 1), with SA and GR resulting more intense. Similar results were confirmed also in terms of safranal flavour, while as regards to the intensity of safranal odour and flavour GR and SA showed the highest levels, respectively. No differences were recorded as related to fruity, flowery and grassy notes, while for other (not defined) odours and flavours, GR was the less intense. CT recorded the highest intensity in astringency and pungency, while no differences were found for bitterness. Starch flavour was less intense in SA.

Table 3 shows the result of crocins perception thresholds related to the perceptions of bitterness and astringency intensity. The test on crocins at different concentrations highlighted that bitter and astringent attributes could be clearly perceived. The bitter recognition threshold (50% of the population) was recorded at 0.94; at the same concentrations, 95% of the assessors perceived astringency. Bitter sensation was always accompanied by astringency.

**Figure 2:**
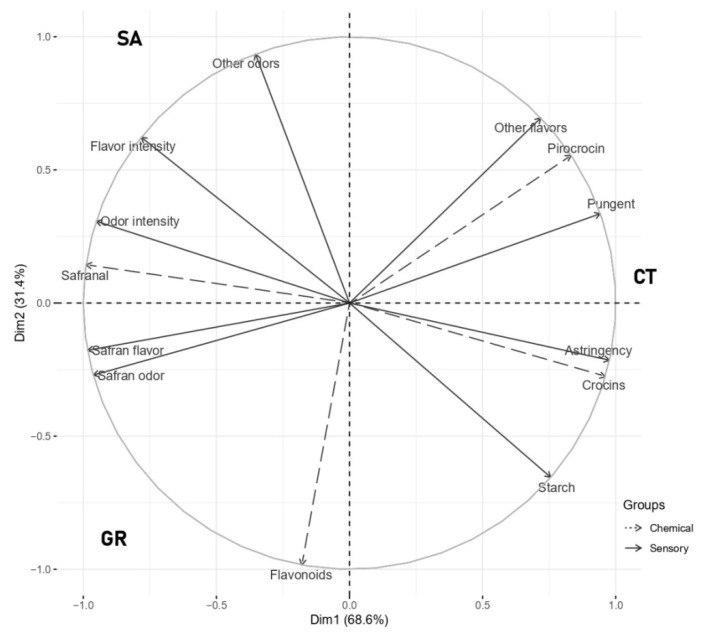
Multiple Factor Analysis map. The map describes correlation between sensory attributes (continuous arrows) and the main saffron compounds chemically analysed (dashed arrows). SA, CT, GR mark the graph dials related to Sardinian, Central Tuscany and Southern Tuscany saffron, respectively.

To elicit possible correlations between the concentrations of main saffron compounds and sensory attributes, a multiple factor analysis (MFA) was performed as shown in Figure 2.

MFA shows that the safranal amount (dashed arrow) is largely correlated to saffron flavour and odour and overall odour intensity, as perceived by judges (continuous arrows). Picrocrocin content (dashed arrow) is also correlated to the perceived pungency (continuous arrow). A clear correlation was found also for crocins (dashed arrow) and astringency (continuous arrow). Flavonoids content (dashed arrow) was not correlated to any of the sensory attributes evaluated.

## 4. Discussion

Saffron quality is based on safranal concentration providing the typical aroma and on picrocrocin bitter taste. In this work, both biochemical and sensory analysis have elicited important differences in the profiles of three Italian saffron samples of different geographical origins. Distinctive characteristics in biochemical composition of the three saffron samples are probably due to specific agro-environmental factors affecting local saffron productions, as in the case of the two Tuscan samples, even though the cultivation areas are relatively close (about 170 km of distance). Significant differences were also perceived when the three types of saffron samples were tasted. However, bitterness, as the saffron key sensory component, was not differently perceived in the three samples by the trained panel. On the other hand, the present work highlights how other crocins can also contribute to bitter intensity and to key sensory attributes such as, astringency and pungency. Specific tests on crocins showed that these compounds, in solution, provide both bitter and astringent sensations at relatively low concentrations.

Although more research will be needed to further integrate the two analytical methods, in our knowledge this is the first report that produces evidence of the correlation between biochemical and sensory data obtained and contributes to define saffron quality and safety traceability. Besides, data on secondary metabolites (responsible for the organoleptic and sensory characteristics) provides important contents for saffron commercial labelling and proper information to consumers.

## Figures and Tables

**Table 1:** Second metabolites content of dried stigmas of SA, CT, GR saffron samples. Average value ± SD (standard deviation)of three samples. Data are expressed as mg/g sample. SA: Sardinia sample, CT: central Tuscany sample, GR: south Tuscany sample.

Saffron Samples			
COMPOUNDS	SA	CT	GR
Crocins			
Trans crocin 5	5.95 ± 0.22	3.46 ± 0.11	1.43 ± 0.03
crocin derivative	2.37 ± 0.06	3.19 ± 0.10	1.74 ± 0.04
crocin derivative	2.71 ± 0.07	2.55 ± 0.05	1.33 + 0.03
trans crocin 4	169.97 ± 2.18	405.19 ± 3.95	249.82 ± 2.92
crocin derivative	2.07 ± 0.06	3.67 ± 0.12	2.06 ± 0.08
trans crocin 3	61.22 ± 1.80	132.07 ± 1.98	93.90 ± 2.15
crocin derivative	3.70 ± 0.16	4.15 ± 0.13	1.44 ± 0.05
trans crocin 2’	-	-	3.74 ± 0.15
crocin derivative	1.18 ± 0.04	0.96 ± 0.02	0.48 ± 0.1
crocin derivative	1.04 ± 0.03	1.12 ± 0.02	-
cis crocin 4	25.91 ± 0.92	24.13 ± 0.09	11.84 ± 0.47
crocin derivative	-	-	0.27 ± 0.1
trans crocin 2	16.26 ± 0.55	26.59 ± 0.93	21.48 ± 0.89
crocin derivative	3.85 ± 0.11	3.51 ± 0.10	1.63 ± 0.06
cis crocin 3	1.96 ± 0.05	1.77 ± 0.07	3.61 ± 1.02
crocin derivative	2.07 ± 0.06	1.44 ± 0.06	1.26 ± 0.05
crocin derivative	-	1.12 ± 0.05	0.27 ± 0.01
crocin derivative	1.18 ± 0.02	1.12 ± 0.05	0.32 ± 0.01
crocin derivative	1.04 ± 0.02	1.12 ± 0.05	0.46 ± 0.01
**TOTAL**	**302.49**	**617.14**	**397.1**
**safranal**	3.11 ± 0.10	0.48 ± 0.01	2.66 ± 0.08
**picrocrocin**	74.33 ± 1.92	112.62 ± 2.01	29.13 ± 1.01
**Flavonoids**			
K-3-sophoriside -7-glucoside	2.86 ± 0.09	3.08 ± 0.10	5.95 ± 0.21
k der	0.89 ± 0.04	-	-
K-3-sophoroside	6.05 ± 0.23	8.91 ± 0.033	10.54 ± 0.42
**TOTAL**	**9.79**	**11.99**	**16.49**

**Table 2:** Nutritional table. Physical parameters, nutritional and chemical element content of dried stigmas of SA, CT, GR saffron samples (see Material and methods). Data are expressed as percentages (physical parameters and nutritional compounds) and mg/kg (chemical elements). Data are the mean of three determinations (standard deviation < 2%).

Sample	Moisture	Ash	Fibres	Proteins	Fat	Sugars	Na	K	Mg	Ca	Fe	Cu	Zn	Mn
**SA**	7.6	8.7	3.8	14.5	4.9	59.2	15.5	13,098	417	811	121.8	10.3	104.2	31.7
**CT**	9.4	11.3	3.8	17.3	5.6	51.3	212.5	14,680	252	99.7	64.1	2.4	25.2	24.3
**GR**	10.8	6.4	3.7	15.2	5.1	57.3	6.25	13,752	1315	1005	57.9	8.1	87.8	28.4

**Table 3:** Crocins are calculated as ppm (parts per million). Threshold scores refer to the attribute intensity of bitterness and astringency at different crocin concentrations.

	***Perception Threshold***	
***Crocins***	***Bitterness Scores***	***Astringency Scores***
0.94	1.9	2.6
1.87	2.4	2.6
3.75	2.2	3
7.5	3	3.2
15	2.8	3.1
30	2.8	3.2
60	3.2	3.5
